# Applying different genomic evaluation approaches on QTLMAS2010 dataset

**DOI:** 10.1186/1753-6561-5-S3-S9

**Published:** 2011-05-27

**Authors:** Javad Nadaf, Ricardo Pong-Wong

**Affiliations:** 1 The Roslin Institute and R(D)SVS, University of Edinburgh, Easter Bush, Midlothian, EH25 9RG, UK

## Abstract

**Background:**

With the availability of high throughput genotyping, genomic selection, the evaluation of animals based on dense SNP genotyping, is receiving more and more attention. Several statistical methods have been suggested for genomic selection. Compared to traditional selection, genomic selection can be more accurate which can lead to higher efficiency in terms of time and cost. Herein we applied different genomic evaluation methods on the 14^th^ QTLMAS dataset.

**Methods:**

Four different approaches were used for the estimation of EBV of animals for the Quantitative and the Binary Trait (QT and BT respectively). It included two Bayes B types of approaches (BB): using only SNP information (GBB) or SNP and Pedigree information (GPBB); and two genomic BLUP, GBLUP and GPBLUP. Traditional BLUP was also used only for comparison. When using BB methodology, the probability of SNP having an effect on the traits (which include a quantitative and a binary trait) were also estimated. We also performed “standard” QTL mapping approaches including linkage and association analyses to compare them with BB results as a potential QTL mapping tools.

**Results:**

For QT, the best accuracy of EBV (correlation between EBVs and TBVs) for young animals, was obtained by BB methods (*r* = 0.68). Genomic BLUP estimations (GBLUP and GPBLUP) were less accurate (*r* = 0.60 and 0.61 respectively). Similar results were obtained for the BT: *r* were estimated at 0.82, 0.82, 0.71 and 0.70 for GPBB, GBB, GPBLUP and GBLUP respectively. Using traditional BLUP, *r* was at 0.39 and 0.47 for QT and BT respectively. The genetic correlation between the two traits (approximated by the correlation between EBVs for BT and QT using GBB method) was as high as 0.58.

**Conclusions:**

Better accuracies were obtained using BB methods, compared to BLUP analyses. Compared to the traditional BLUP, the accuracy of the EBVs was improved about 70% and 50% using BB and GBLUP methods respectively. The benefit of genomic selection was the same for both the QT and BT. Models with and without polygenic effect led to similar accuracies in the estimation of breeding values. The BT and QT were genetically correlated (*r*=0.58) which suggested that bivariate analyses may be of advantages. Signal profile by GBB followed well the true QTL patterns, which was consistent with good estimation of EBVs by this method, suggesting its potential value for QTL mapping.

## Background

Genomic selection can be described as the use of highly dense genotyping in the evaluation of animals, to increase the accuracy of the estimated breeding values (EBV) [1]. Several statistical methods has been suggested and applied. Roughly speaking, they can be grouped into two categories. In the first group, the effects of all SNP in the map are jointly estimated, and then the EBV for each animal is calculated as the sum of all SNP effects, given their genotype. Meuwissen et al [1] compared several methods using this approach and the best performance was achieved when the model accounted for the fact that not all SNP in the map are affecting the trait (i.e. Bayes B, BB). This method also allows estimating the probability of a SNP having an effect on the trait, which can be used as a criterion for QTL mapping. In the second group, SNP genotype is used to better estimate the relationship among individuals [2]. The benefit of this is that, such estimations can be later used in a standard Best Linear Unbiased prediction analysis (GBLUP) to calculate EBV. The advantages from using this approach are its speed and the availability of software, as the mixed model theory is well-established.

Regardless the approach used for genomic selection, their success would depend on the quality of the SNP map to capture the whole genetic variation, which would depend on several factors such as Linkage Disequilibrium (LD) between loci and the coverage of the whole genome. In order to safeguard against possible problems related to the quality of the SNP panel, the model can be modified to include an extra genetic effect which is explained by the pedigree information. A model combining both source of information may prove to be beneficial.

The aim of this study was to compare the results evaluations from using these two methods (a modified Bayes B and genomic BLUP) with and without polygenic in the model to evaluate animals in the QTLMAS dataset. We also compared BB results with “standard” association and linkage analyses, to assess its potential values for QTL mapping.

## Methods

The data used is the simulated dataset distributed by the organisers of the QTLMAS workshop 2010. The population consists of 3226 individuals spanning 5 generations, of which the last 900 individuals have no phenotype for a quantitative and a binary trait (QT and BT). Genome is about 500 Mb long distributed in 5 chromosomes. All individuals have genotype for 10031 SNPs.

### 1. Genomic evaluation

#### a. Bayes B type models

Bayes B (BB) method was first described by Meuwissen et al.[1]. Basically, this type of method assumes that only a proportion of the SNP (π) is affecting the trait. We applied a modified approach with two differences Firstly, if the SNP is affecting the trait, its effect is normally distributed with the same variance. Secondly, the proportion π is estimated from the data, rather than assuming to be known *a priori*. Applying these assumptions, two models, with (GPBB) or without polygenic effects (GBB), were fitted:

where, **y** is the vector of phenotypes; µ is the population mean for the trait; n is total number of SNP, **z**_i_ is the vector of genotypes at SNP i; *β_i_* indicates the allelic substitution effect for SNP i; and **e** is the vector of residuals. The allelic substitution effects *β* for each SNP were assumed to come from a mixture distribution, with probability of π to have a non-zero effect on the trait, , and with probability of (1- π) of not affecting the trait; **a** is the genetic addictive effect explained by pedigree information and assumed to be normally distributed, , where **A** is the additive relationship matrix calculated from pedigree information,  is the variance of the effect explained by the pedigree. **Z** is the incidence matrix. Here, thereafter, the effect associated to the pedigree will be referred as the polygenic effects.

The models were implemented using Gibbs sampling. The parameters π and  were estimated from data using flat priors. For each analysis, a MCMC chain was run and the first 10000 cycles were discarded as burn-in period. Following this, 50000 realisations were collected, each separated by 20 cycles between consecutive realisations (i.e. length of chain = 1,010,000 cycles). The posterior mean was used as the estimate for each parameter of interest.

For the binary trait a liability threshold model was used.

In order to estimate the relative value of the genetic effect explained by the SNP or the pedigree information, the genetic variance explained by genomic information (SNP) was estimated. The simplest approach would have been to sum the variance individually explained by each SNP given their effects and frequencies, but this may be biased because it does not account for the LD between loci. In order to avoid this problem, an approximation based on the infinitesimal model theory was used [3]:

PEV stands for Prediction Error Variance, and *r* is the accuracy of estimates. The explained additive genetic variance () was obtained using the above equations, for BB methods and the corresponding proportion of this variance (to the total variance) was reported.

#### b. Genomic BLUP models

The genomic BLUP consists in using SNP genotype to estimate the relationship between individuals which later are used into the mixed model. Two genomic BLUP models, with and without polygenic effect, were fitted:

where, **g** is vector of random additive genetic effect explained by the SNP information and assumed to be normally distributed as , where **G** is the realised relationship matrix calculated from SNP information [4], and  is the variance of **g**. Both GBLUP models were implemented using ASREML [5], where the variance components were estimated from the data itself. For the binary trait, the logit link function was used [5].

#### c. Model comparisons

The main criterion of comparison between the different genomic approaches was using the correlation between the total estimated breeding values (which includes the polygenic effect associated with the pedigree if added into the model) and the true breeding values (TBV). Alternatively, within each method we compared the model with and without polygenic effect using Bayes Factor (BF) [6] and likelihood ratio test (LRT) for the BB and GBLUP, respectively.

### 2. QTL mapping

Additionally to the estimated SNP effects, BB methods also estimate the probability of SNP having an effect on the trait, which can be used as a criterion for QTL mapping. In order to assess its potential in use, we compared these results with standard association and linkage analyses.

#### a. Association analyses

Association analyses were performed using the GRAMMAR approach [7], which comprises two steps. First, phenotype were corrected for the polygenic effects and second, residuals were fitted against each SNP using additive model as implemented in GENABEL[8]. The binary trait was treated as a quantitative trait.

#### b. Linkage analyses

##### - Haf-sib QTL mapping

Half-sib analyses (HS-QTL) were performed as described by Haley et al. [9], and implemented in the GridQTL [10]. The analysis was based on studying the segregation of the paternal allele. The binary trait was treated as quantitative one.

##### - Variance component QTL mapping

As the population is distributed across several generations creating a complex pedigree structure, a QTL mapping based on a variance component approach (VC-QTL) may perform better than the mapping based on half sibs regressions. Here, we performed this analysis for the quantitative trait. The method is based on a two-step approach [11]: At each position, first, a relationship matrix based on Identical-By-Descent (IBD) coefficients was estimated using a recursive method [12]. Then a REML analysis was performed to calculate the variance components. Likelihood Ratio Test (LRT) was used as the test statistics to compare the model with QTL versus the one without QTL[13].

## Results and discussion

### Genomic evaluation

Correlations between TBV and EBVs estimated by the different methods were shown in Table 1. Bayes B methods (GBB and GPBB) had the highest accuracies for both QT (0.68) and BT (0.82). GBLUP methods were less accurate, with about 0.60 and 0.71 for QT and BT, respectively. Table 1 also showed the results of using the traditional BLUP model where the genetic effect was estimated based on the pedigree information only. Traditional BLUP was performed using the Bayesian and frequentist approach when compared to the BB and GBLUP results, respectively. Compared with traditional BLUP, using genomic information improved the accuracy of the EBVs by about 70% (BB models) and 50% (GBLUP models).

**Table 1:** Correlation between true and estimated breeding values of unphenotyped individuals for the different genomic methods.

Methods	Quantitative Trait	Binary Trait
GBB	0.679	0.823

GPBB	0.678	0.824

GBLUP	0.604	0.714

GPBLUP	0.607	0.714

Traditional BLUP*	0.391	0.471

*Results from traditional frequentist BLUP were added for comparison.

Table 1 also showed that the inclusion of the effect associated with the pedigree has little impact on the accuracy of the EBV of unphenotyped individuals. This result was unexpected for the quantitative trait, because the analysis including this extra genetic effect showed that it explained approximately a third of the total genetic variance (Table 2). This result was consistent in both the BB and GBLUP models. Moreover, the model comparison test using Bayes Factors (BB) or LRT (GBLUP) showed that adding this extra genetic component makes the model to fit better the data (results not shown). This difference in the partition of the genetic variance, however, was not reflected on the accuracy of EBV for unphenotyped animals. The addition of genetic effect associated to the pedigree information was not important on the analysis of the binary trait (Table 3).

**Table 2:** Heritability estimates for the quantitative trait using different genomic methods.

		polygenic	SNP(genomic)	Total
BB	GPBB	16	40	56
	
	GBB	-	47	47
	
	Traditional BLUP*	55	-	55

BLUP	GPBLUP	15	36	51
	
	GBLUP	-	42	42
	
	Traditional BLUP*	54	-	54

* Results for traditional BLUP are from the Bayesian and frequentist approaches when compared with BB and BLUP, respectively.

**Table 3:** Heritability estimates for the Binary trait using the different genomic methods.

		Polygenic	SNP	Total
BB*	GPBB	5	45	50
	
	GBB	-	46	46
	
	Traditional BLUP^$^	43	-	43

BLUP*	GPBLUP	~0	36	36
	
	GBLUP	-	36	36
	
	Traditional BLUP^$^	19	-	19

* Two different models (Logit link function or liability threshold) were used for BLUP and BB respectively (see text for more details) which may make the results between the two methodologies less comparable.

^$^ results for traditional BLUP are from the Bayesian and frequentist approaches when compared with BB and BLUP, respectively.

**Figure 1:**
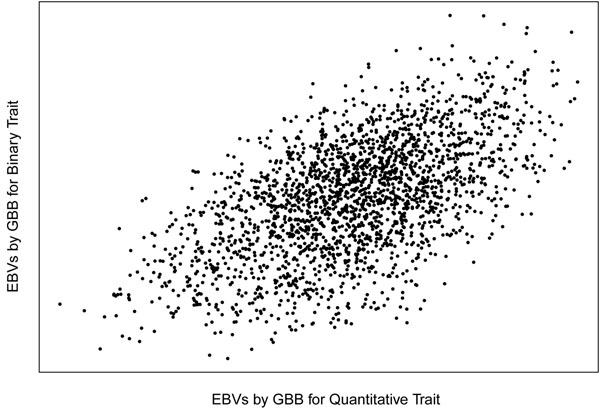
Plot of EBVs for the BT and QT using GBB method (r2 = 0.58).

Correlation between EBV for both traits was around 0.58 across the four genomic selection methods (Figure 1). This result was expected as the simulation assumed 30 QTL affecting QT, and 22 of them were also affecting BT. BB method estimated the proportion of SNP having an effect on the trait to be 0.9% and 1.8% for the QT and BT, respectively. Hence, the BB approach needed to use approximately 90 and 180 linked SNP to explain the whole genetic variance explained by these QTL.

### QTL mapping

**Figure 2:**
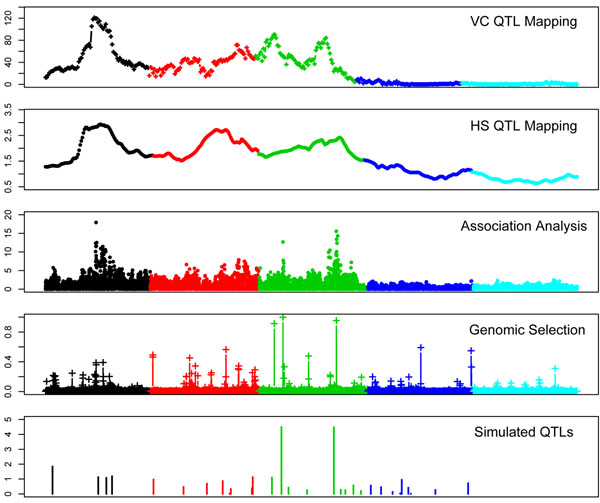
Comparison of QTL mapping profiles: linkage analyses, association and genomic selection (GBB) for quantitative trait. Different colours mean different chromosomes (1 to 5). The scales on y axes from top to down are: LRT (Likelihood Ratio Test); F statistic, F statistic, probability (of having effect) and simulated substitution effect.

**Figure 3:**
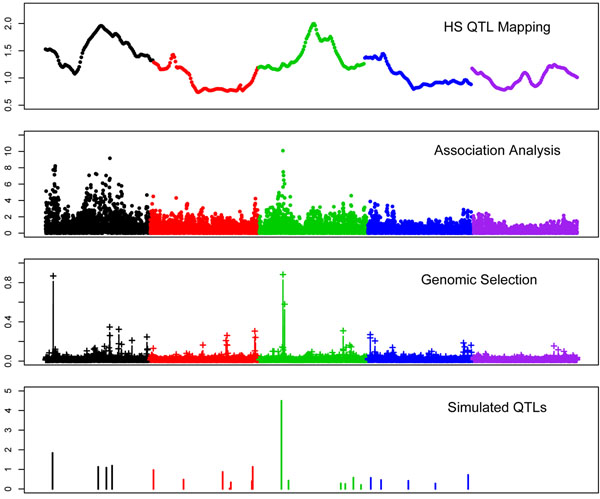
Comparison of QTL mapping profiles: linkage analyses, association and genomic selection (GBB) for binary trait. Different colours mean different chromosomes (1 to 5). The scales on y axes from top to down are: F statistic, F statistic, probability (of having effect) and simulated substitution effect.

BB estimated the probability of a given SNP having an effect on the trait. Locating single SNP or a cluster of linked SNP with a relatively large probability may be used as criteria for mapping QTL. Figures 2 and 3 compare the QTL mapping profiles obtained by the association, linkage analyses and GBB with the profile of the true simulated QTL. The comparison between methods was based on the similarity in the trend of the profiles regardless they were significant or not, and to test for consistence between methods rather than their power for detecting QTL. In general the linkage, association and BB analyses were consistent on the position for the largest QTL affecting traits. However, there were some differences worth to be noticed. For the QT, the strongest signals of the presence of a QTL found with linkage and association methods (VC-QTL, HS-QTL and association) was on chromosome 1, but the SNP with the highest probability found with GBB was on chromosome 3, which was consistent with the simulated QTL. Additionally, GBB found signals of possible QTL on chromosomes 4 and with lesser extent on chromosome 5, which all were missed by the linkage and association analyses. Positions on chromosome 4 were consistent with actual simulated QTL patterns but those on chromosome 5 were false positives. Similarly good consistency also was observed for the BT (Figure 2). The most important point to mention is the profiles on chromosome 3. The chromosome included few QTL on the proximal and several small QTL at its distal part, which was similar with the QTL profile obtained by BB, and also association analysis. Instead, linkage analysis appeared to detect a ghost peak in the middle of the chromosome. Generally speaking, the signal profile observed by GBB (measured as the probability of the SNP having an effect on the trait) followed the true QTL pattern.

The good performance of the BB, in terms of genomic evaluation of animals, was also reflected in the consistent QTL signals, obtained by the method, compared to the actual simulated QTLs, raising its potential value for QTL mapping.

## Competing interests

The authors declare that they have no competing interests.

## Authors' contributions

JN and RPW carried out the analyses and drafted the manuscript. Both authors have read and contributed to the final text of the manuscript.
